# Changes in liver-related mortality by etiology and sequelae: underlying versus multiple causes of death

**DOI:** 10.1186/s12963-021-00249-0

**Published:** 2021-04-29

**Authors:** Ming-Jen Sheu, Fu-Wen Liang, Ching-Yih Lin, Tsung-Hsueh Lu

**Affiliations:** 1 Division of Gastroenterology and Hepatology, Chi Mei Medical Center, Tainan, Taiwan; 2 Department of Medicinal Chemistry, Chia Nan University of Pharmacy and Science, Tainan, Taiwan; 3 Department of Public Health, Kaohsiung Medical University, Kaohsiung, Taiwan; 4 Department of Public Health, College of Medicine, National Cheng Kung University, Tainan, Taiwan

**Keywords:** Mortality, Cause of death, Underlying cause of death, Multiple causes of death, Burden of disease, Liver disease, Hepatitis C virus, Alcoholic liver disease, Cirrhosis, Primary liver cancer, Secondary liver cancer

## Abstract

**Background:**

The expanded definition of liver-related deaths includes a wide range of etiologies and sequelae. We compared the changes in liver-related mortality by etiology and sequelae for different age groups between 2008 and 2018 in the USA using both underlying and multiple cause of death (UCOD and MCOD) data.

**Methods:**

We extracted mortality data from the CDC WONDER. Both the absolute (rate difference) and relative (rate ratio and 95% confidence intervals) changes were calculated to quantify the magnitude of change using the expanded definition of liver-related mortality.

**Result:**

Using the expanded definition including secondary liver cancer and according to UCOD data, we identified 68,037 liver-related deaths among people aged 20 years and above in 2008 (29 per 100,000) and this increased to 90,635 in 2018 (33 per 100,000), a 13% increase from 2008 to 2018. However, according to MCOD data, the number of deaths was 113,219 (48 per 100,000) in 2008 and increased to 161,312 (58 per 100,000) in 2018, indicating a 20% increase. The increase according to MCOD was mainly due to increase in alcoholic liver disease and secondary liver cancer (liver metastasis) for each age group and hepatitis C virus (HCV) and primary liver cancer among decedents aged 65–74 years.

**Conclusion:**

The direction of mortality change (increasing or decreasing) was similar in UCOD and MCOD data in most etiologies and sequelae, except secondary liver cancer. However, the extent of change differed between UCOD and MCOD data.

## Background

**Fig. 1:**
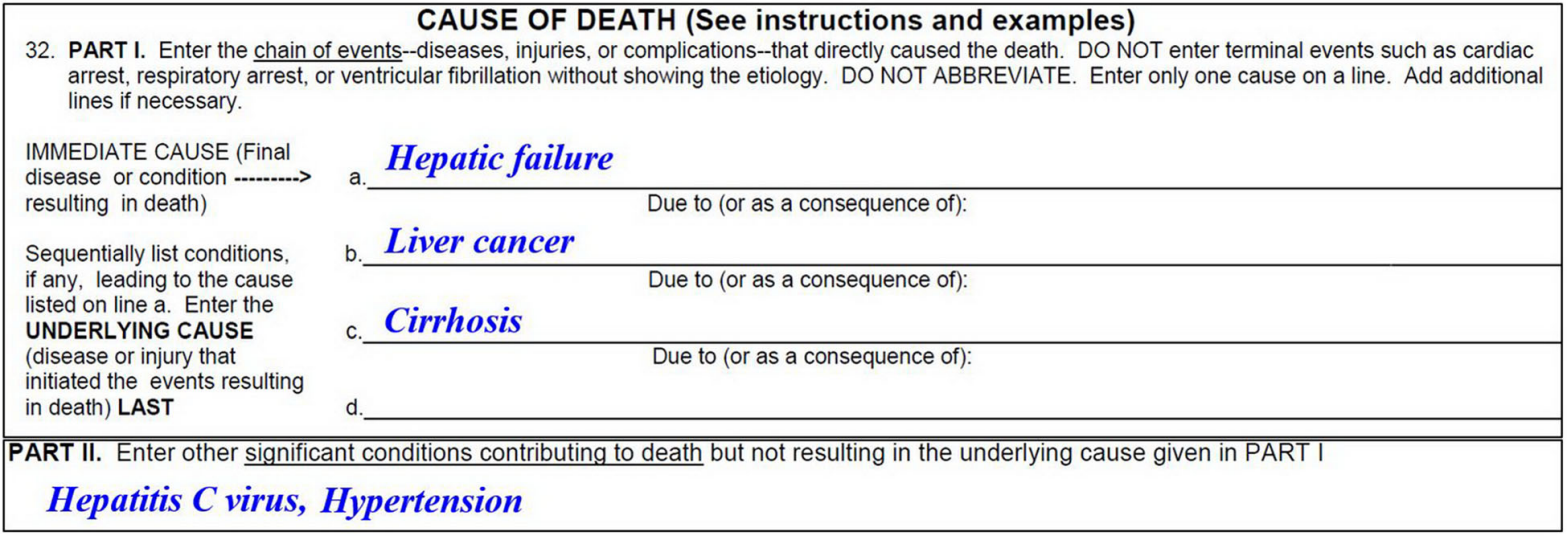
The certifying physician recorded four liver-related diagnoses on the death certificate. Only “liver cancer” would be selected as the underlying cause of death for mortality tabulation

Cause of death mortality data are the most complete and standardized population-based health data that can be used to estimate the burden of health problems at a national level. Changes in cause-specific mortality can be examined to assess the effectiveness of interventions and identify emergent health problems. Several studies have examined the changes in liver-related mortality in the USA (e.g., hepatitis C virus infection [HCV] [1–4], viral hepatitis [5], cholestatic liver disease [6], alcoholic liver disease [7, 8], nonalcoholic fatty liver disease [9], liver cancer [10], chronic liver disease [11], and cirrhosis and liver cancer) [12, 13]. However, these studies only investigated one specific liver disease, which does not provide a comprehensive representation of the complexity of liver-related mortality, because certifying physicians may record several liver-related diagnoses on death certificates (Fig. 1). Several scholars have suggested using an expanded definition of liver-related deaths, which includes a wide range of etiologies (hepatitis B or C virus infection, or alcoholic or toxic liver disease) and sequelae (liver cancer, cirrhosis, esophageal varices, hepatorenal syndrome, or hepatic failure), to more accurately assess liver-related mortality burden [14–19].

The National Center for Health Statistics’ official published mortality data are compiled based on the underlying cause of death (UCOD), which is defined as “(a) the disease or injury which initiated the train of morbid events leading directly to death, or (b) the circumstances of the accident or violence which produced the fatal injury” [20]. If the certifying physician records several liver-related diagnoses, such as HCV in part 2 and cirrhosis, liver cancer, and hepatic failure in part 1 of the death certificate (Fig. 1), then “liver cancer” would be selected as the UCOD according to the international selection rules set by the World Health Organization and thus would be categorized as a liver cancer death in mortality data [20]. Numerous scholars have suggested using multiple cause of death (MCOD) data to make full use of information provided by the certifying physicians [19, 21–28].

Studies have reported a decline in HCV associated mortality since 2014 [4, 11], and a persistent increase in mortality from alcoholic liver disease [8, 11], cirrhosis, and liver cancer [12, 13]. However, mortality changes have not been presented by etiology and sequelae for different age groups using both UCOD and MCOD data. Furthermore, previous studies did not include secondary liver cancer (liver metastasis) in the expanded definition. The diagnosis and treatment of liver metastasis require relatively high-cost and multidiscipline inputs (e.g., surgical, medical, radiation, and interventional specialties), which should be included in the estimation of mortality burden of liver-related mortality [29, 30]. This study sought to compare the changes in liver-related mortality by etiology and sequelae for different age groups from 2008 to 2018 in the USA using UCOD and MCOD data.

## Methods

UCOD and MCOD mortality data were extracted from the Center for Disease Control and Prevention Wide-ranging Online Data for Epidemiologic Research (CDC WONDER) for 2008 and 2018 [31]. The International Statistical Classification of Diseases and Related Health Problems, Tenth Revision (ICD-10) codes for the expanded definition of liver-related deaths are based on those in the study of Asrani et al. [17], as illustrated in Table 1.

**Table 1:** ICD-10 codes for liver-related disease

Liver-related disease	ICD-10 codes
Hepatitis C virus	B171, B182
Other hepatitis	B15, B16, B170, B172, B178, B179, B180, B181, B188, B189, B19, K73
Primary liver cancer	C22
Secondary liver cancer	C787
Esophageal varices	I85
Alcoholic liver disease	K70
Hepatic failure	K72
Liver cirrhosis	K74
Other diseases of liver (toxic, inflammatory, and others)	K71, K75, K76

*ICD-10* International Statistical Classification of Diseases and Related Problems Tenth Revision

Decedents aged 20 years and over were included. Age-specific liver-related mortality rates were calculated for the age groups of 20–44, 45–64, 65–74, and ≥75 years, and age-standardized death rates (ASDR) were calculated using the age structure of the US population for 2000 as the standard population. Both the absolute (rate difference) and relative (rate ratio) changes and 95% confidence intervals (95% CI) were calculated to quantify the magnitude of change from 2008 to 2018 for overall and specific liver-related mortality rates.

These calculations were performed using UCOD and MCOD data separately. The decedent in Fig. 1 would be categorized as one liver cancer death using the UCOD approach, whereas they would be categorized as one death with HCV, one death with cirrhosis, one death with liver cancer, and one death with hepatic failure if the MCOD approach was used. However, for overall liver-related deaths in MCOD data, the decedent in Fig. 1 would be counted as one death only. The percentage of UCOD/MCOD deaths was calculated to reflect the contribution of MCOD in the increase in the number of deaths.

## Results

Using the expanded definition including secondary liver cancer and according to UCOD data, we identified 68,037 liver-related deaths among people aged 20 years and above in 2008 (ASDR was 29 per 100,000) and this increased to 90,635 in 2018 (ASDR was 33 per 100,000), a 13% increase from 2008 to 2018. However, according to MCOD data, the number of deaths was 113,219 (ASDR was 48 per 100,000) in 2008 and increased to 161,312 (ASDR was 58 per 100,000) in 2018, indicating a 20% increase. If we excluded secondary liver cancer, the percentage of increase was 14%, from 42 per 100,000 (98,657 deaths) in 2008 to 48 per 100,000 (133,216 deaths) in 2018.

**Fig. 2:**
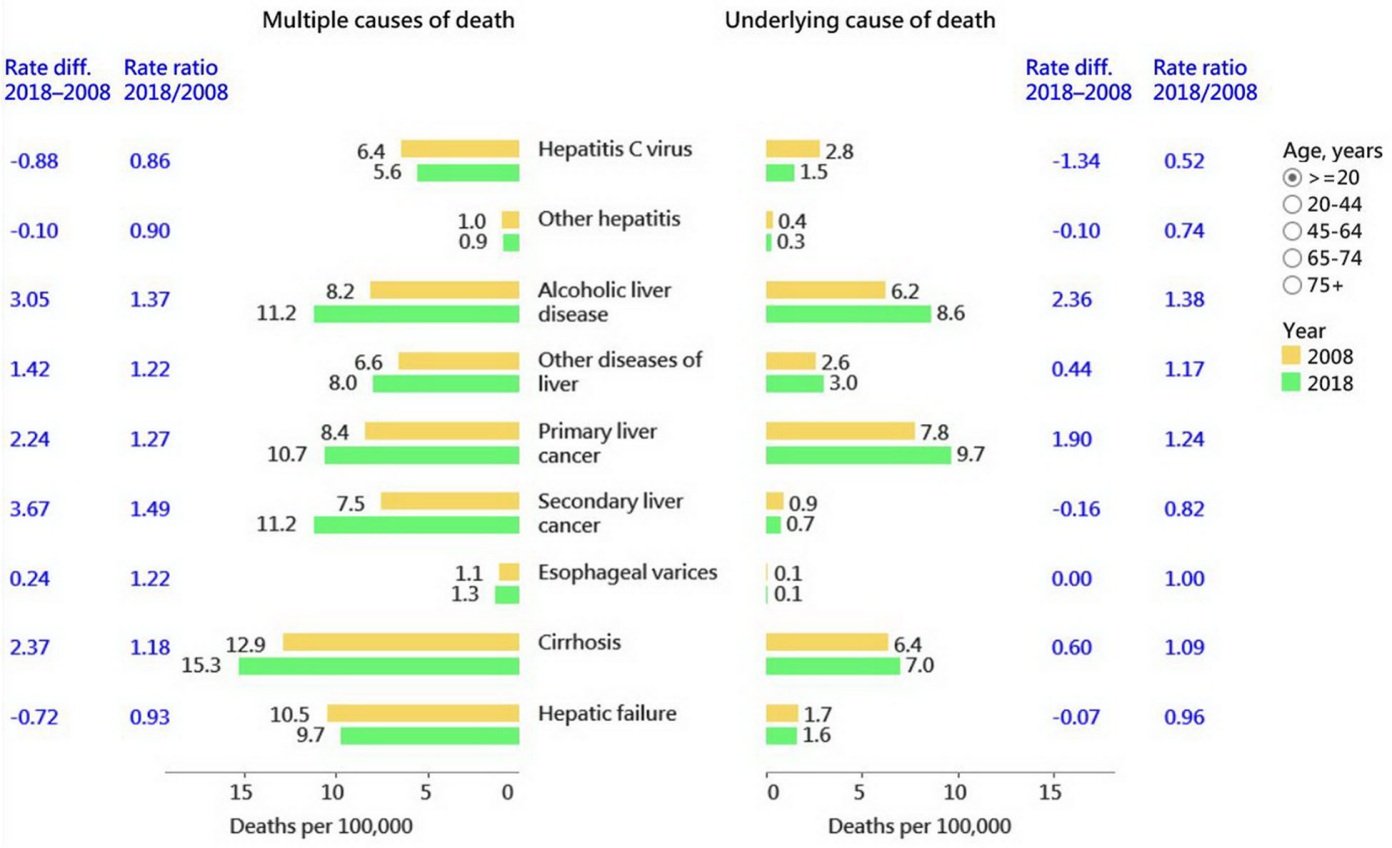
Absolute (rate difference) and relative (rate ratio) change in the expanded definition of liver-related mortality rates (deaths per 100,000) from 2008 to 2018 according to underlying cause of death versus those based on multiple cause of death data in the USA. The users can select the age group they are interested in the following data visualization dashboard. https://public.tableau.com/profile/robert.lu#!/vizhome/20072017USA/Liver-relatedmortality

The rate differences and rate ratios from 2008 to 2018 for overall and specific etiology and sequelae are summarized in Table 2 and Fig. 2. Mortality associated with HCV exhibited the largest decline according to both UCOD and MCOD data, followed by other hepatitis and hepatic failure. Mortality associated with alcoholic liver disease exhibited the largest increase, followed by primary liver cancer. Mortality from secondary liver cancer (liver metastasis) decreased according to UCOD data but increased according to MCOD data. The UCOD/MCOD percentage was the highest for primary liver cancer (92% in 2008 and 91% in 2018), followed by alcoholic liver disease (76% in 2008 and 76% in 2018), and was the lowest for esophageal varices (6.9% in 2008 and 5.8% in 2018), followed by secondary liver cancer (11.9% in 2008 and 6.6% in 2018).

**Table 2:** Age-standardized death rate of liver-related disease in 2008 and 2018 according to underlying and multiple cause of death (UCOD and MCOD) data

	2008		2018		Rate		
Liver-related disease	Deaths	Rate	Deaths	Rate	Difference	Ratio	(95% CI)
Overall							
UCOD	68,037	28.8	90,635	32.5	3.6	1.13	(1.11–1.13)
MCOD	113,219	48.2	161,312	57.6	9.5	1.20	(1.18–1.20)
UCOD/MCOD, %	60.1		56.2				
Overall (excluding secondary liver cancer)							
UCOD	66,175	28.0	88,785	31.8	3.80	1.14	(1.12–1.14)
MCOD	98,657	41.8	133,216	47.7	5.88	1.14	(1.13–1.14)
UCOD/MCOD, %	67.1		66.6				
**Etiology**							
Hepatitis C virus							
UCOD	6834	2.8	4127	1.5	−1.3	0.52	(0.50–0.54)
MCOD	15,707	6.4	15,712	5.6	−0.9	0.86	(0.84–0.88)
UCOD/MCOD, %	43.5		26.3				
Other hepatitis							
UCOD	883	0.4	755	0.3	−0.1	0.74	(0.66–0.80)
MCOD	2280	1.0	2381	0.9	−0.1	0.90	(0.84–0.94)
UCOD/MCOD, %	38.7		31.7				
Alcoholic liver disease							
UCOD	14,864	6.2	23,171	8.6	2.4	1.38	(1.35–1.40)
MCOD	19,530	8.2	30,446	11.2	3.1	1.37	(1.34–1.39)
UCOD/MCOD, %	76.1		76.1				
Other diseases of liver							
UCOD	6077	2.6	8507	3.0	0.4	1.17	(1.13–1.20)
MCOD	15,394	6.6	22,228	8.0	1.4	1.22	(1.19–1.24)
UCOD/MCOD, %	39.5		38.3				
**Sequelae**							
Primary liver cancer							
UCOD	18,159	7.8	27,647	9.7	1.9	1.24	(1.22–1.26)
MCOD	19,666	8.4	30,441	10.7	2.2	1.27	(1.24–1.28)
UCOD/MCOD, %	92.3		90.8				
Secondary liver cancer							
UCOD	2071	0.9	2104	0.7	−0.2	0.82	(0.76–0.86)
MCOD	17,341	7.5	31,676	11.2	3.7	1.49	(1.46–1.51)
UCOD/MCOD, %	11.9		6.6				
Cirrhosis							
UCOD	14,993	6.4	19,615	7.0	0.6	1.09	(1.07–1.11)
MCOD	30,463	12.9	43,165	15.3	2.4	1.18	(1.16–1.20)
UCOD/MCOD, %	49.2		45.4				
Esophageal varices							
UCOD	181	0.1	211	0.1	0.0	1.00	(0.80–1.19)
MCOD	2619	1.1	3663	1.3	0.2	1.22	(1.15–1.27)
UCOD/MCOD, %	6.9		5.8				
Hepatic failure							
UCOD	3975	1.7	4498	1.6	−0.1	0.96	(0.91–0.99)
MCOD	24,578	10.5	27,059	9.7	−0.7	0.93	(0.91–0.94)
UCOD/MCOD, %	16.2		16.6

The age differences in rate differences and rate ratios from 2008 to 2018 for overall and specific etiology and sequelae are reported in Table 3. For alcoholic liver disease, we noted increase in each age group according to both UCOD and MCOD. For secondary liver cancer increase in each age group occurred only in MCOD not in UCOD. For primary liver cancer, the increase confined to decedents aged 65–74 years old in both UCOD and MCOD. For HCV, only decedents aged 65–74 years old showed increase change according to MCOD only. The figures of mortality changes by age could be viewed in https://public.tableau.com/profile/robert.lu#!/vizhome/20072017USA/Liver-relatedmortality.

**Table 3:** Liver-related disease death rate difference (RD) and rate ratio (RR) and 95% confidence intervals (95% CI) between 2008 and 2018 according to underlying and multiple cause of death (UCOD and MCOD) data

	20-44 years			45-64 years			65-74 years			75+ years		
Liver-related disease	RD	RR	(95% CI)	RD	RR	(95% CI)	RD	RR	(95% CI)	RD	RR	(95% CI)
Overall												
UCOD	0.52	1.11	(1.06–1.15)	2.89	1.06	(1.04–1.08)	18.24	1.29	(1.26–1.31)	9.65	1.12	(1.09–1.13)
MCOD	0.98	1.13	(1.09–1.15)	9.38	1.14	(1.12–1.14)	37.26	1.34	(1.31–1.35)	32.15	1.21	(1.19–1.22)
Overall (excluding secondary liver cancer)												
UCOD	0.54	1.11	(1.07–1.15)	2.81	1.06	(1.04–1.07)	18.59	1.31	(1.27–1.33)	11.48	1.15	(1.12–1.17)
MCOD	0.56	1.07	(1.04–1.10)	4.69	1.07	(1.06–1.08)	28.44	1.31	(1.28–1.33)	18.67	1.16	(1.13–1.17)
**Etiology**												
Hepatitis C virus												
UCOD	−0.26	0.37	(0.30–0.43)	−3.43	0.47	(0.44–0.48)	−0.21	0.94	(0.85–1.03)	−1.54	0.54	(0.46–0.60)
MCOD	−0.31	0.68	(0.61–0.74)	−3.74	0.75	(0.72–0.76)	6.24	1.75	(1.65–1.85)	−1.64	0.77	(0.71–0.83)
Other hepatitis												
UCOD	−0.01	0.86	(0.60–1.11)	−0.19	0.69	(0.59–0.77)	−0.10	0.85	(0.65–1.03)	−0.28	0.71	(0.55–0.86)
MCOD	−0.03	0.88	(0.72–1.04)	−0.36	0.79	(0.72–0.84)	0.34	1.20	(1.04–1.35)	−0.08	0.96	(0.82–1.09)
Alcoholic liver disease												
UCOD	0.90	1.43	(1.35–1.51)	4.45	1.36	(1.32–1.39)	4.33	1.43	(1.35–1.50)	1.49	1.31	(1.19–1.41)
MCOD	1.05	1.40	(1.33–1.47)	5.41	1.33	(1.29–1.35)	6.87	1.52	(1.45–1.58)	2.64	1.40	(1.30–1.50)
Other diseases of liver												
UCOD	−0.10	0.82	(0.71–0.91)	−0.09	0.98	(0.92–1.02)	2.42	1.41	(1.31–1.51)	3.60	1.49	(1.38–1.58)
MCOD	0.03	1.02	(0.94–1.08)	0.64	1.07	(1.03–1.09)	6.32	1.45	(1.38–1.50)	7.66	1.43	(1.37–1.49)
**Sequelae**												
Primary liver cancer												
UCOD	0.01	1.03	(0.89–1.15)	1.74	1.19	(1.15–1.22)	10.03	1.50	(1.44–1.55)	5.31	1.15	(1.11–1.18)
MCOD	0.00	1.00	(0.87–1.13)	2.11	1.21	(1.17–1.24)	11.37	1.53	(1.47–1.57)	6.56	1.17	(1.13–1.21)
Secondary liver cancer												
UCOD	−0.02	0.46	(0.20–0.71)	0.05	1.08	(0.95–1.21)	−0.25	0.89	(0.78–0.99)	−1.78	0.69	(0.62–0.75)
MCOD	0.31	1.56	(1.40–1.72)	4.73	1.67	(1.61–1.72)	9.87	1.44	(1.39–1.49)	13.96	1.38	(1.33–1.42)
Cirrhosis												
UCOD	0.01	1.01	(0.91–1.10)	0.62	1.07	(1.03–1.10)	1.91	1.12	(1.06–1.16)	2.79	1.13	(1.08–1.17)
MCOD	0.00	1.00	(0.93–1.06)	1.77	1.09	(1.06–1.11)	10.97	1.35	(1.30–1.38)	9.97	1.25	(1.21–1.29)
Esophageal varices												
UCOD	−0.01	0.74	(0.28–1.19)	0.01	1.11	(0.80–1.40)	0.01	1.05	(0.54–1.55)	−0.01	0.93	(0.49–1.37)
MCOD	0.01	1.04	(0.88–1.19)	0.36	1.17	(1.09–1.24)	0.96	1.58	(1.37–1.77)	0.39	1.24	(1.05–1.41)
Hepatic failure												
UCOD	0.00	1.00	(0.84–1.14)	−0.28	0.89	(0.83–0.94)	0.12	1.03	(0.93–1.12)	0.06	1.01	(0.92–1.10)
MCOD	0.06	1.03	(0.96–1.09)	−1.23	0.92	(0.89–0.94)	−1.03	0.96	(0.92–0.99)	−3.35	0.89	(0.85–0.92)

The users can select the age group they are interested in the data visualization dashboard.

## Discussion

This national population-based study compared the changes in liver-related mortality by etiology and sequelae among different age groups from 2008 to 2018 in the USA using the expanded definition (including secondary liver cancer) and according to both UCOD and MCOD data. The direction of mortality change (increasing or decreasing) was similar in UCOD and MCOD data in most etiologies and sequelae, except secondary liver cancer. However, the extent of change differed between UCOD and MCOD data. The magnitude of decreasing changes in HCV mortality was more prominent in UCOD data than MCOD, particularly for decedents aged 65–74 years (baby boomers born 1945–1965). However, the extent of increasing changes was more drastic in MCOD data than in UCOD data for esophageal varices, cirrhosis, and other diseases of the liver. The magnitude of increasing changes in UCOD and MCOD data was similar for primary liver cancer and alcoholic liver disease.

Kim et al. [11] examined four etiology-based mortality changes according to UCOD and MCOD data. Their study indicated a mild reduction of mortality for the hepatitis C virus infection from 2007 to 2014 according to UCOD data, with an annual percentage change (APC) of −0.4%, followed by a prominent decreasing trend in the APC of −13.7% from 2014 to 2016. A different pattern of change was observed using MCOD data; the APC was 2.0% from 2007 to 2014 and −6.4% from 2014 to 2016. For alcoholic liver disease, the magnitude of mortality changes was similar according to UCOD and MCOD data; the APC was 5.3% and 5.5% from 2014 to 2016, respectively. Kim et al. further examined mortality changes from 2007 to 2016 for liver-related sequelae (i.e., cirrhosis and liver cancer) and observed APCs of 2.3% and 2.0%, respectively [13].

We extended the study of Kim and colleagues to examine mortality changes by four age groups. We observed a huge increase in mortality in HCV (rate ratio was 1.75 with 95% CI 1.65–1.85) and primary liver cancer (rate ratio was 1.53 with 95% CI 1.47–1.57) according to MCOD that occurred only in decedents aged 65–74 years old and not in other age group. This age specific change was mainly due to the aging of baby boomers [32].

Another new finding of this study is the 50% increase in the number of deaths from secondary liver cancer (liver metastasis) according to MCOD data, from 17,346 in 2008 to 31,689 in 2018. The liver is the most common site for gastrointestinal tumor metastasis because of the mesenteric venous outflow through the portal vein. The most common liver metastasis is colorectal cancer, which is the third leading cancer cause of death in the USA. Approximately, 15 to 20% of patients with colorectal cancer have synchronous liver metastases at presentation and 50% eventually develop liver metastases. Metastatic disease in the liver is also commonly observed in lung cancer, neuroendocrine tumors, gastrointestinal stromal tumors, breast cancer, gastric cancer, melanoma, and pancreatic cancer [29, 30, 33–35]. However, no study has used mortality data to examine the changes in liver metastasis mortality. Further studies are needed to clarify the epidemiology of liver metastasis-associated mortality, including the distribution of original sites.

One of the strengths of this study is the examination of age differences in changes in liver-related mortality according to traditional definition versus expanded definition. The second strength was the addition of secondary (metastatic) liver cancer in the expanded definition. The third strength was the use of data visualization dashboard in which the users can select the mortality pattern of particular age group they are interested in. The fourth strength was the use of both UCOD and MCOD, which provided a more comprehensive overview of the complexity of liver-related mortality.

Several limitations should be considered when interpreting the findings of this study. First, studies that have examined the information recorded on the death certificate with medical records have suggested underreporting of certain liver-related etiologies and sequelae on the death certificate [16–18]. However, as our primary aim was to examine the changes in liver-related mortality, a systemic bias between 2008 and 2018 caused by underreporting is unlikely. The recommendation of HCV screening in 2012 [36] and the introduction of DAA in 2013 would have increased the reporting of HCV by certifying physicians on the death certificate from 2008 to 2018. Therefore, the extent of decline of recordings of comorbid HCV and liver cancer or cirrhosis among baby boomers from 2008 to 2018 estimated in this study would be underestimated. That to say, the true magnitude of decline would be larger than we estimated.

Second, to avoid complexity in presentation, we did not examine all combinations among different etiologies and sequelae. According to the study of Ly et al., there were more than 20 combinations among five etiologies, including hepatitis B and C virus, alcoholic liver disease, and nonalcoholic steatohepatitis/fatty liver disease [18]. Including five liver-related sequelae (primary and secondary liver cancer, cirrhosis, esophageal varices, and hepatic failure) would result in just under 100 combinations. In this study, we presented only two crucial combinations (i.e., HCV with liver cancer and HCV with cirrhosis). Third, to avoid the complexity of presentation, we did not analyze data for each year from 2008 to 2018, and we did not further analyze differences in sex and ethnicity. Fourth, information on the severity of sequelae is not available on the death certificate. Some people with esophageal varices or liver metastasis might have required large amounts of medical care resources, whereas others did not. There are numerous modules in the treatment of liver metastasis, with large variations in costs; thus, using mortality data alone cannot accurately estimate the healthcare resources used. Fifth, there have been age-period-cohort effects on cirrhosis and liver cancer mortality, the use of only 2 years and ASDR might obscure the differences that may be of interest.

## Conclusion

Despite the abovementioned limitations, we can firmly conclude that presenting the mortality changes using both UCOD and MCOD data by etiology and sequelae and by age could provide a more comprehensive overview of the complexity of liver-related mortality, particularly for baby boomers (born during 1945–1965), because they exhibit a high prevalence of HCV. The findings of this study indicated a decline in mortality associated with HCV from 2008 to 2018, particularly among baby boomers, suggesting an effect of the change in screening recommendations in 2012 and the introduction of DDA in late 2013.

## Data Availability

The datasets used and/or analyzed during the current study are available from the corresponding author on reasonable request.

## Abbreviations

CDC WONDER: The Center for Disease Control and Prevention Wide-ranging Online Data for Epidemiologic Research
HCV: Hepatitis C virus
ICD-10: The International Statistical Classification of Diseases and Related Health Problems, Tenth Revision
MCOD: Multiple causes of death
UCOD: Underlying cause of death
